# Why we need to be careful with LLMs in medicine

**DOI:** 10.3389/fmed.2024.1495582

**Published:** 2024-12-04

**Authors:** Jean-Christophe Bélisle-Pipon

**Affiliations:** Faculty of Health Sciences, Simon Fraser University, Burnaby, BC, Canada

**Keywords:** artificial intelligence, AI ethics, LLM, medicine, AI regulation, AI governance, AI deregulation, hallucination

## Introduction

Large language models (LLMs), the core of many generative AI (genAI) tools, are gaining attention for their potential applications in healthcare. These applications are wide-ranging, including tasks such as assisting with diagnostic processes, streamlining patient communication, and providing decision support to healthcare professionals. Their ability to process and generate large volumes of text makes them promising tools for managing medical documentation and enhancing the efficiency of clinical workflows (1). LLMs offer a distinct advantage in that they are relatively straightforward to use, particularly since the introduction of ChatGPT-3.5, and they exhibit a notable alignment with human language and communication patterns, facilitating more natural interactions (2) and acceptance of the LLMs' conclusions (3). LLMs operate by predicting the next word in a sequence based on statistical correlations identified in large datasets (4, 5). However, while these models are effective at producing text that appears coherent and contextually appropriate, they do so without a genuine understanding of meaning or context. This limitation is particularly significant in healthcare, where accuracy is critical. Unlike human cognition, which is driven by a complex array of goals and behaviors, LLMs are narrowly focused on text generation. This focus can lead to the production of plausible sounding but inaccurate information, a phenomenon referred to as “AI hallucination” (6). In high-stakes environments like prediction, triaging, diagnosis, monitoring, or patient care, these inaccuracies can have serious consequences.

While numerous articles across various *Frontiers* journals discuss LLMs, relatively few focus on AI hallucinations as a central issue. For example, Jin et al. (35) in *Frontiers in Medicine* note that “While LLMs like ChatGPT offer tremendous potential in ophthalmology, addressing the challenges of AI hallucination and misinformation is paramount.” Similarly, Giorgino et al. (34) in *Frontiers in Surgery* emphasize that “The responsible use of this tool must be based on an awareness of its limitations and biases. Foremost among these is the dangerous concept of AI hallucination.” Beyond the realm of healthcare, Williams (38) in *Frontiers in Education* observes that “The concept of AI hallucination gained widespread attention around 2022, coinciding with the rise of LLMs such as ChatGPT. Users noticed these chatbots often generated random falsehoods in their responses, seemingly indifferent to relevance or accuracy.” Williams (38) continues by stressing that the “term AI hallucination has been criticized for its anthropomorphic connotations, as it likens human perception to the behavior of language models.” Despite these critical discussions, they remain sparse compared to the many articles praising LLMs in medicine, highlighting the need for greater engagement in addressing the limitations of these technologies. This imbalance highlights the need for greater emphasis on mitigating the risks posed by these models. Building on this concern, Hicks et al. (10) challenge conventional thinking in their paper “ChatGPT is Bullshit.” They assert that the inaccuracies produced by LLMs should not simply be labeled as “hallucinations,” but as “bullshit,” a term based on philosopher Frankfurt's (7) work. According to this perspective, “bullshit” reflects a disregard for accuracy, which poses serious challenges for the use of genAI in healthcare. By reconceptualizing LLMs in healthcare as “bullshiting” instead of “hallucinating,” this paper aims to provide a perspective on the risks these tools pose in critical applications. It explores practical solutions such as layered LLM architectures and improved XAI methods, and emphasizes the urgency of implementing tailored oversight mechanisms to counterbalance the political and industry push for AI deregulation in sensitive domains like medicine.

## Understanding AI's “bullshit”

LLMs generate text by predicting the next word based on large datasets. While they produce human-like text, they don't inherently understand or verify its accuracy, acting as “prop-oriented make-believe tools” (8). Their errors are not the result of technical glitches that can be resolved with better data or refined algorithms but stem from their fundamental nature—they do not evaluate evidence or reason in the human sense. This critical distinction between LLMs' statistical processing and human reasoning can lead to misconceptions, particularly when LLMs are portrayed or perceived as capable of human-like cognition. While LLMs can generate accurate and contextually relevant text, their outputs are based on statistical correlations, not genuine comprehension. As Bender et al. (32) famously argued, LLMs, which generate word sequences based on learned patterns, function as “stochastic parrots.” In contrast, human reasoning involves deeper cognitive processes such as understanding, critical thinking, and interpretation. While some, like Downes et al. (33), challenge this view, suggesting that LLMs can produce sensible answers by leveraging higher-level structural information inherent in their design, the fact remains that LLMs remain fundamentally agnostic to empirical reality. Recognizing this distinction is crucial, as the statistical predictions made by AI models—no matter how convincing—should not be equated with deliberate, evidence-based reasoning of the human mind. As Hicks et al. (10) point out: “ChatGPT is not trying to communicate something they believe or perceive. Their inaccuracy is not due to misperception or hallucination. As we have pointed out, they are not trying to convey information at all. They are bullshitting.” This indifference to evidence is especially concerning in medicine, where accuracy, interpretability, and liability are paramount. Consider the implications of using genAI to provide medical advice or assist in diagnosing patients—if the nature of its outputs is misunderstood, it poses significant risks. Trusting and acting on potentially flawed information could result in misdiagnoses and improper treatments, with serious consequences for patient care. As stated by Harrer (1): “Health buyers beware: generative AI is an experimental technology not yet ready for primetime.”

Recognizing that these AI systems produce “bullshit” rather than “hallucinations” calls for a more cautious and skeptical approach, according to Hicks and colleagues. Titus (23) convincingly stated that “Attributing semantic understanding to these systems when we are not warranted in doing so could have serious social and ethical implications related to anthropormorphizing (sic) these systems or over-trusting their ability to produce meaningful or truthful responses.” In the health sector, this implies that, medical professionals should be wary about them and avoid using LLMs as standalone sources of information or advice (9). If AI systems are inherently indifferent to the truth, there is a heightened responsibility on developers and users to ensure these tools do not cause harm. This involves not only improving the technical accuracy of AI models but also clearly communicating their limitations to users. As Hicks et al. (10) note, “Calling chatbot inaccuracies ‘hallucinations' feeds into overblown hype about their abilities among technology cheerleaders, and could lead to unnecessary consternation among the general public. It also suggests solutions to the inaccuracy problems which might not work, and could lead to misguided efforts at AI alignment amongst specialists.” Given the significant ethical implications of AI in medicine, LLMs should be used as supplementary tools with expert validation of both medical AI design and outputs prior to clinical applications (9, 11).

Ensuring AI trustworthiness in healthcare requires shared responsibility, with developers creating transparent systems and medical professionals critically assessing AI outputs and their limitations (12–15). Medical professionals must be trained to understand that AI-generated content that may sound convincing, is not always reliable. Developers should prioritize creating interfaces that highlight these limitations and encourage critical evaluation of AI outputs. For example, including disclaimers or confidence scores can help users better assess the reliability of the information provided (16). This is basically what the Notice and Explanation section of the White House's AI Bill of Rights (17) requires: “Medical professionals should not use AI as a standalone source of information or advice. Instead, AI should serve as a supplementary tool, with all outputs rigorously validated by human experts before being applied in any clinical setting.” However, disclosure is not enough in itself as it is also conducive to problems, particularly by shifting the burden onto users. Such disclosure should be accessible and understandable in a way that does not reproduce the problems of consumer products' *Terms and Conditions*, which are made ridiculously long to ensure that nobody reads them (18).

## Could more LLMs be the solutions?

Employing multiple layers of LLMs to mitigate the limitations inherent in individual models could be a way to solve the previously raised issues. Work is currently underway in this area (19). Usually this entails enabling one model to cross-validate the outputs of another to identify and correct inaccuracies, thereby reducing the incidence of AI hallucination. This layered approach, wherein different models are assigned specialized tasks such as fact-checking or contextual validation, has the potential to enhance the robustness and reliability of AI-generated content (20). However, this methodology introduces significant complexity, including the risk of error propagation and the challenges associated with the coordination of multiple models. Furthermore, while this strategy, which Verspoor (36) calls “fighting fire with fire,” may incrementally improve the accuracy of outputs, it fails to address the foundational issue of LLMs' lack of true semantic understanding. An over-reliance on layered LLMs could result in diminishing returns, where the added complexity and potential for novel errors negate the anticipated benefits of enhanced accuracy. Additionally, this approach risks fostering an overdependence on AI systems (21), potentially undermining the role of human expertise in domains requiring nuanced understanding and ethical decision-making.

LLMs can still offer valuable contributions to medical practice if used wisely. LLMs can assist in administrative tasks, generate patient documentation, or provide preliminary information on medical topics. They can even be useful in defending patients' interests in health insurance claims (22). However, these applications must be designed with safeguards to prevent over-reliance on potentially inaccurate outputs (9). One way to enhance LLMs' utility in medicine is not to rely solely on them, but also to implement verification systems based on reliable databases (not just web-scrapping). Even Hicks et al. (10) emphasize that there are practical solutions to address the concerns of AI “bullshit.” For example, connecting a LLM to a trusted medical database can help ensure the information it provides is cross-referenced with reliable sources. Such a system would also incorporate a mechanism for arbitrating evidence, further enhancing accuracy and providing a certain level of trustworthiness. However, this integration must be implemented carefully to avoid introducing new forms of misinformation or inadvertently embedding values that are inconsistent with the context in which the tool is being deployed (11).

## Could explainable AI and regulatory frameworks solve the problem?

Explainable AI (XAI) aims to increase transparency in AI decision-making, including in LLMs. Techniques like attention mechanisms and *post-hoc* explanations help users understand how AI generates outputs, especially in high-stakes fields like healthcare. However, XAI does not address the core limitation: LLMs depend on statistical patterns, not genuine reasoning or evidence evaluation (23). Moreover, while these techniques are valuable for tracing outputs back to their underlying processes, they often fail to expose the deeper epistemic limitations of LLMs, such as their inability to reason or evaluate evidence. Their explanations, therefore, reflect these patterns rather than any meaningful understanding. Regulatory frameworks, such as the European Union's AI Regulation (24) and the US AI Bill of Rights Blueprint (17), establish critical standards for transparency, safety, and accountability. However, adapting LLMs to meet these standards may not overcome their fundamental limitations in reasoning and evidence-based decision-making. Experts argue for shifting focus from refining LLMs to developing new AI paradigms, such as neurosymbolic AI, which combines neural networks with logical reasoning to address these gaps.

Neurosymbolic AI offers a promising alternative, integrating neural adaptability with logical precision to enable more robust reasoning and contextual understanding (25, 26). These models can potentially overcome key limitations of LLMs, offering greater efficiency and interpretability. As Wadhwa (37) suggests, LLMs are nearing their developmental ceiling, and further investment in them risks diminishing returns. Instead, regulators and investors may explore advancing neurosymbolic AI to drive the next generation of innovation, while ensuring AI systems are both transparent and capable of increased trustworthy reasoning. Despite its promise, neurosymbolic AI is not a panacea. It faces challenges in scalability, interpretability, and handling the complexity of real-world medical data (27). Moreover, its reliance on logical structures may not fully capture the nuances of probabilistic and ambiguous information common in medicine. Thus, while neurosymbolic AI represents an incremental advance, robust oversight, multidisciplinary collaboration, and continued innovation remain essential for addressing AI's limitations in critical domains like healthcare.

## Discussion

A deep, critical examination of the inherent limitations of LLMs is crucial for advancing medical AI in ways that prioritize patient safety and ethical integrity. While LLMs like ChatGPT can generate fluent, coherent text, this proficiency often conceals a more troubling reality: their responses are not necessarily grounded in verified facts or consistent logic. In the medical field, where evidence-based decision-making is paramount, relying on these models without addressing their fundamental flaws presents significant risks. LLMs, at their core, are probabilistic models designed to predict the next word in a sequence based on patterns in training data. This mechanism, though powerful for generating human-like text, is fundamentally indifferent to truth. If the goal of the model is to generate the most statistically likely response rather than the correct or most appropriate one, there is a significant risk of misinformation infiltrating clinical workflows.

As Jin et al. (35) underscore, “Responsible AI implementation and continuous monitoring are essential to harness the benefits of AI while minimizing potential risks.” A key concern with LLMs in medical applications is their lack of reproducibility. Unlike traditional software systems, where identical inputs yield consistent outputs, LLMs can generate different answers to the same question on different occasions. This unpredictability undermines the reliability needed in medical settings, where consistency is essential for delivering safe and effective care. Medicine, as a discipline, cannot afford to embrace tools that exhibit *epistemic insouciance*—a disregard for the reliability and validity of knowledge. This is especially problematic given that LLMs, in many cases, are not anchored in factual reality but are designed to produce text that merely sounds plausible. The use of the term “hallucination” to describe when LLMs generate factually incorrect statements trivializes the severity of the issue. In truth, this behavior reflects a deeper problem: LLMs are trained to predict patterns, not to produce factual outputs. In medicine—an evidence-based practice since the 1990s—this fundamental flaw can lead to the adoption of unreliable tools that compromise the integrity of patient care.

The standard disclaimers provided by models like ChatGPT, which warn that “ChatGPT can make mistakes. Check important info,” are insufficient safeguards in clinical settings. While Harrer (1) points out that “In defense of OpenAI, it never advertised ChatGPT as trustworthy advisor but rather as a crowdsourced technology evaluation and refinement experiment”; Harrer also acknowledged that there is insufficient risk mitigation across genAI, including ChatGPT, which has sparked growing caution amid internet-level hype. The implications for the health sector are significant, most users (especially healthcare professionals) lack the time or expertise to verify every piece of AI-generated information, especially in high-stake environments where the margin for error is slim, but the consequences significant. Entrusting users with the responsibility of fact-checking AI outputs without giving them the resources or assurances of accuracy exposes the field to potentially dangerous mistakes, as well as to arguably lead to AI ethics dumping, so to offload such responsibility to downstream users (28). The casual acceptance of these limitations in AI use—particularly in medicine, where errors can have life-threatening consequences—reflects a dangerous complacency. Transparency, interpretability, and trustworthiness in medical AI are not a luxury but a necessity. Healthcare professionals need to understand not only what the AI recommends but also how and why it arrived at its conclusions. Explainability in AI systems is critical for building trust and enabling professionals to make informed decisions based on AI output. Without this transparency, the tools are “black boxes,” offering answers without accountability or justification—an untenable situation in clinical decision-making.

The challenges of ensuring ethical and trustworthy AI are further amplified by the current political climate, especially in the United States. The incoming Trump administration is expected to prioritize the removal of “unnecessary” AI regulations to accelerate innovation (29). The lobbying efforts of influential tech organizations like BSA | The Software Alliance (30)—which represents companies such as OpenAI and Microsoft—advocate for policies that reduce regulatory constraints to promote AI adoption. While the group acknowledges the importance of international governance and standards, its focus on removing barriers to innovation risks deprioritizing critical safeguards (such as government-imposed ethical AI standards and oversight mechanisms). Furthermore, President-elect Trump's plans to undo AI regulatory efforts by the previous administration—including a risk management framework designed to foster AI transparency and accountability—signal a potential shift toward AI deregulation (31), and perhaps an AI regulation winter. Such a move could weaken efforts to mitigate the inherent risks of deploying LLMs and flawed AI systems in high-stakes domains like healthcare.

Given this context, it is crucial to emphasize shared responsibility for trustworthy AI systems. Developers, policymakers, and healthcare institutions must collaborate to uphold ethical standards, transparency, and accountability in AI deployment, regardless of the regulatory environment. Without such efforts, the drive for deregulation may exacerbate the risks posed by LLMs, particularly their tendency to produce plausible yet inaccurate or misleading outputs. Trustworthy AI cannot be treated as a secondary consideration, especially in healthcare, where patient outcomes and lives are directly at stake.

Reframing AI errors from being seen as harmless “hallucinations” to recognizing them as dangerous “bullshit” is more than just a shift in terminology—it is a critical reframing of how to approach the integration of AI into healthcare. These are not small, occasional mistakes but fundamental flaws in how these systems operate. Policymakers, healthcare providers, and AI developers must recognize that the stakes are high, and that without rigorous safeguards, LLMs and genAI could erode trust and the quality of care.
